# Challenging Health Citizenship: Digital Health Engagement Among the Oldest Age Groups

**DOI:** 10.1111/1467-9566.70042

**Published:** 2025-11-11

**Authors:** Hanna Varjakoski, Fredrika Thelandersson, Amalie Søgaard Nielsen

**Affiliations:** ^1^ Department of Social Sciences University of Eastern Finland Kuopio Finland; ^2^ Department of Communication Lund University Lund Sweden; ^3^ Department of Design, Media and Educational Science University of Southern Denmark Odense Denmark

## Abstract

This article investigates how older citizens perceive and utilise digital health technologies as part of their everyday health management, health‐related risk monitoring and illness prevention. This study draws on empirical data collected as part of the HAIDI research project and include interviews and media go‐alongs with 71 individuals aged 75–100 years living in Sweden, Finland and Denmark. Interviews were conducted in 2023, and the transcribed data were analysed using a mix of inductive and deductive approaches. Four overarching themes emerging from the data are explored in the context of late life, reflecting the complexities and nuances of health maintenance and practices in a landscape increasingly shaped by digital health: aiming for self‐responsibilisation, utilising digital health technologies for health/risk management, challenging promises of digital health and negotiating health expertise. In this study, the accomplishment of digital health citizenship in late life appeared selective and fluctuating but often also relational, which challenges the existing individualist theorisations of digital health citizenship. This study produces new knowledge on how the oldest citizens navigate the digital health landscape and critically examines the evolving concept of digital health citizenship in the context of ageing, late life and healthcare.

## 1 Introduction

One morning, 76‐year‐old Lasse sat at his kitchen table, carefully wrapping the familiar cuff around his arm. He had become accustomed to this daily ritual, dutifully taking his own blood pressure at home. The small monitor beeped and hummed, its numbers flashing predictably on the screen. However, today, something was different. The numbers were alarmingly high, more than they had ever been. Worried, he decided to visit his local health clinic. The nurse there, seeing the unusually high readings, quickly sent him to the emergency room at the nearest hospital. There it was discovered that Lasse was suffering from atrial fibrillation—a heart condition he had never known he had.

The example above conveys how contemporary citizens have taken an active role in managing their health and in taking preventive measures. Digital health technologies are said to enable individuals to track their health indicators and well‐being, access health information and engage in digital health consultations, for instance, with healthcare providers when needed. It is argued that the utilisation of health‐related digital devices, applications and resources generates ‘autonomous and responsible citizens’ (Bavngaard 2023, 30), as these tools facilitate self‐care and health monitoring that foster self‐responsibility in health and risk management. And, as Petersen (2019) points out, digital technologies are simultaneously also reshaping conceptions of citizenship, self and society, which further relate to broader discourses of digital competence as a key citizenship skill (Ilomäki et al. 2016; Lupton 2013).

Because of the growing proportion of older adults, the Nordic countries' reliance on digital health has become prominent. Digital health can be defined in several ways, including both how healthcare systems operate and how individuals engage with technology for their health (Petersen 2019). With efforts to increase (cost‐)efficiency in the health sector, there is an increasing expectation that citizens will use digital health technologies and services to manage their personal health. In Nordic countries, digital services have already become the primary choice for healthcare and social services, where national strategies for digitalising healthcare aim to create active and self‐directed citizens who take responsibility for their own health management (Ministry of Social Affairs and Health 2024, 14). This follows a longer trend in Western countries where the neoliberal reconfiguration of the welfare state has entailed increased individual responsibility for managing health and illness since the start of the 21st century (Rose 2007). The digitalisation of health has only emphasised this shift and highlighted that individuals are no longer mere recipients of health services and users of related technologies but must enact a normatively and ideologically defined digital health citizenship (Petrakaki et al. 2021).

As the key targets for healthcare provision by digital technologies and as the largest user group of healthcare services, older people are among those most affected by the digitalisation of health (Poli et al. 2023, 2265). Given this focal remark related to the older demographic, this article focuses on older adults and health citizenship in a landscape increasingly shaped by digital health technologies and services. Existing studies have already indicated that older adults use different digital health resources, technologies and services (see, e.g., Sturm et al. 2023; Bavngaard 2023; Grønning 2021), yet not much is known about the oldest age groups' engagement with digital health citizenship. In addition, studies on digital technology and older people have tended to focus on technologies explicitly dedicated to care, whereas less attention has been given to everyday health technologies (Hirvonen et al. 2022). Therefore, in this article, we ask: *How do older adults aged 75 and above perceive and utilise digital health technologies as part of their everyday health management, health‐related risk monitoring and illness prevention?*

We approach the concept of digital health citizenship as a sociopolitical ideal that is open to sociological critique and argue that discussions about (digital) health citizenship would benefit from critically exploring the abilities of different social groups, such as older adults, to participate in and enact health citizenship—particularly if they face challenges related to health and digitalisation. Instead of focusing on one specific technology or digital service, we have adopted a broad and bottom‐up approach where our interviewees' experiences have informed us what is included in the category of digital health. This approach adds to the uniqueness of this study and allows us to see our interviewees as agents who, in their everyday living and reasoning, actively negotiate a health citizenship increasingly shaped by digitalisation.

Our study produces new knowledge on the oldest citizens' (75+) everyday (dis)engagement with digital technology in a health context, a demographic that can be hard to reach and is often overlooked in digital health‐related theorisation. We start by outlining the theoretical background of this study and previous research on the topic, followed by a description of the methodological approach. After this, we present the results and the concluding discussion.

## 2 From Biological Citizenship to Digital Health Citizenship

In contemporary Western neoliberal society, ideal citizens are expected to actively inform themselves about current diseases, manage potential risks and take appropriate steps to maximise health and minimise illness by adjusting lifestyle through acts of choice. Individuals are urged to be flexible, train and learn continuously, and monitor themselves to constantly optimise their health (Rose 2007). Rose and Novas (2005) introduced the concept of biological citizenship to highlight how citizenship has been shaped by certain vital characteristics and been the target of medical practices since the 18th century. What marks the biological citizenship of the 21st century are increased pressures on the individual to be responsible for their own health. This is especially visible in European and Australasian welfare states, which previously managed the population's health services but which have been increasingly dismantled since the start of the 21st century (Rose 2007).

Related to and expanding on the notion of biological citizenship is the concept of ‘health citizenship’ (Jauho and Helén 2023), where the individual's vital features, capabilities and potential are used as criteria to assess their membership in a society, to define the quality of their citizenship and to allow or restrict their access to citizenship entitlements. Health citizenship recognises that health is not solely determined by genetics or medical interventions but is influenced by individual actions, social contexts and digital technologies. Citizens are seen as co‐producers of health, urged to make informed decisions about lifestyle, preventive measures and self‐care. Jauho and Helén (2023, 479) note that this has made personal healthcare a morally loaded obligation, resulting in both a ‘heightened sense of personal responsibility over one's own health and more vocal blaming of those who are perceived as having failed to fulfil their obligations’. This logic ignores that members of vulnerable and disadvantaged groups may lack capabilities and resources to optimise health, make informed choices or lead a lifestyle seen as healthy. Older adults can be one such vulnerable group that potentially lacks these resources, a lack that becomes heightened with the ongoing digital transition of healthcare services and personal health management.

To acknowledge the digitalisation of healthcare, Petrakaki et al. (2021) have introduced the concept of ‘digital health citizenship’. They argue that the use of health applications, wearable devices and eHealth produces a specific digital health citizenship where individuals are expected to generate data for health‐related purposes, develop expertise in their health condition and actively engage in their health management by using different digital health tools. Crucially, digital health technologies are not only facilitators of health citizenship, but they also produce a set of responsibilities and rights associated with this digitally connected subjectivity. Ideally, digital health can empower individuals to actively engage in their own health management and become co‐producers of health knowledge rather than passive recipients. By using health applications, wearable devices and online platforms, individuals allegedly gain agency over their well‐being (see Petrakaki et al. 2021). However, as will be shown in our findings, this does not mean that citizens willingly aspire to and enact digital health citizenship in the intended ways.

## 3 Health Citizenship in Later Life

In the context of older adults, there is a strong emphasis on healthy ageing, which highlights the importance of regular exercise, healthy eating habits and personal responsibility for maintaining one's health (see, e.g., Sowa et al. 2016). Although people above 75 are a very heterogeneous group in terms of health status (Santoni et al. 2015), older bodies have traditionally been perceived as at‐risk bodies (Katz 2015) and, therefore, been subject to preventative health strategies and imperatives to ‘govern the ageing body’ (Pack et al. 2019). For instance, Tulle (2015) shows how narratives around physical activity in later life represent disciplinary power or Rose's ‘vital politics’ (2007), where particular norms of living are urged upon older adults and sedentary behaviour, in particular, is constructed as a major health risk and a public health concern (also Palmer et al. 2021).

Activity is generally rendered as a universal good in old age and constructed as an inextricable part of healthy ageing (Katz 2000). Illustrative of this is the abundance of national and international guidelines and recommendations that instruct older citizens on how much physical exercise is needed for good health (see, e.g., WHO 2020), how to lead a healthy lifestyle in later life and, consequently, how to enact ‘good’ health citizenship (see also Spoel et al. 2014). Additionally, part of becoming a risk‐aware individual who participates in health politics entails internalisation of the cultural conceptions of the ageing body as being in constant need of monitoring and risk prevention, as exemplified by Kampf's (2010) study on ageing men and prostate cancer.

As the responsibility for health maintenance has shifted to the individual, older adults have been increasingly encouraged to take up digital technologies and applications for self‐monitoring of health and engagement in a healthy lifestyle. There has been growing interest in measuring physical (in)activity to help prevent age‐related problems and to motivate older adults to be ‘tracked and fit’ (Katz and Marshall 2018). Or, as Lolich and Timonen (2021, 36) argue, older adults are becoming ‘ageing entrepreneurs’ who are expected to engage in ageing labour by using digital technologies for self‐care and participate in producing solutions to the challenges brought by ageing, both on individual and public sector levels.

Although older adults still show relatively low levels of fitness tracker, smartwatch and health‐related application use (Seifert and Vandelanotte 2021), the availability of inexpensive home monitoring devices, such as digital blood pressure and blood sugar monitors, has offered new opportunities to do health citizenship and to engage with one's own health maintenance. Measuring and recording one's blood sugar or blood pressure at home is said to help older citizens become informed patients who take more responsibility for their health, concurrently as home self‐care devices provide means for preventative disease monitoring (e.g., Cassarino et al. 2021). Studies indeed have illustrated the diversity of self‐management practices of older individuals living with a chronic illness and the ways in which ‘engaged self‐managers’, in particular, demonstrate responsible self‐governance and independence in managing their long‐term condition (Moore et al. 2015).

Shifting responsibilities from health institutions to individuals has also meant that ‘homes’ have turned into spaces of health monitoring and self‐care, where older persons' use of health devices may rely on family members, and the ‘emplacement’ of devices often mediates the (non)use of health technologies (Weiner and Will 2018). Older individuals also creatively utilise everyday technologies to accomplish healthy ageing, as Loe (2015) has shown, while actively appropriating and rejecting technologies in the process. These notions draw attention to the social, material and technological contexts where older individuals enact and negotiate health citizenship.

## 4 Data and Methods

This study is part of the *Demography and Democracy—Healthy Ageing in a Digital World* (HAIDI) research project and uses datasets from Sweden, Finland and Denmark. The data were collected in 2023 through semi‐structured interviews and media go‐alongs with 71 older citizens (27 in Sweden, 24 in Finland and 20 in Denmark) aged 75–100 years old. Forty‐four of the interviewees were women and 27 men; 47 lived alone and 24 with a spouse. In Finland, the interviewees were recruited via advertisement in a free newspaper; in Sweden, via senior meeting points, personal and professional networks and snowball sampling; and in Denmark, mainly through local care institutions, including nursing homes and home care services.

The interviewees formed a heterogeneous group of individuals above the age of 75. We reached both urban‐ and rural‐dwelling older citizens, individuals with low‐ and high‐income levels, with various educational and work‐life backgrounds and with different health statuses. Our sample consisted of individuals whose first language was Swedish, Finnish or Danish and who possessed the literacy skills necessary to comprehend most communications from the healthcare system and other sources.

The interview guide and the media go‐along protocol were developed jointly among the three authors to ensure common ground for the research. The interview themes included questions related to the interviewees' use or nonuse of digital media technologies and health technologies, health applications and related services, health behaviour and day‐to‐day self‐care. The interviewees were asked, for instance, if they used a smartphone/tablet/computer to interact with healthcare or to find health information and if they used their device for any other health‐related activity. The interviewees were given room to talk about the themes freely, and topics brought up by the interviewees were also included in the data. The interviews were conducted in the interviewees' place of residence (private home, nursing home, assisted living facility), apart from five. One interview was conducted in a local library, two in senior meeting points, one at the university and one via Teams. All the interviewees were interviewed individually, apart from six who were interviewed as couples. The interviews lasted between 60 and 160 min, including the media go‐along protocol, and some (6) were followed up with additional visits by the researcher. The conversations were audio‐recorded with the permission of the interviewee and transcribed verbatim by the authors and professional transcription services. Quotes have been translated from the original language into English by the authors for the purpose of collaborative analysis.

The qualitative media go‐along method (Jørgensen 2016), where participants actively engage with media, enabled us to sharpen our ethnographic approach by observing how interviewees act and react in a specific (constructed) situation instead of only talking about the situation (Kusenbach 2003). In our adapted media go‐along protocol, we constructed scenarios for the interviewees to conduct (using media or not), which the researcher followed along. The scenarios included specific questions such as ‘can you show me how you book an appointment for a vaccination’ and open tour invitations such as ‘can you show me what you use the national health platform for’. Going along with the interviewees in their use of digital health gave the researcher a deeper understanding of the interviewees' actions in specific use cases and considerations during the interview.

The qualitative analysis of the data was conducted with a mix of inductive and deductive approaches (Rivas 2012; Seale 2018). The analytical process was guided by the specific framework of digital health citizenship (Petrakaki et al. 2021), describing how citizens of contemporary neoliberal societies are expected to manage and engage with their health in particular digitalised ways. We sought to understand how this conceptualisation takes shape in the context of old age, yet we also allowed new and opposing themes to arise, as not all managed their health in the ways expected within digital health citizenship.

In the first phase of the analysis, the transcribed data were read several times to obtain a sense of the whole dataset. In the second phase, the software programme NVivo 14 was utilised to organise and code the data. After the coding process, codes and extracts understood as relevant to digital health citizenship were collated and discussed among the three authors. In the next phase, codes were sorted into categories. In the final phase of the analysis, four overarching themes were formed: aiming for self‐responsibilisation, utilising digital health technology for health/risk management, challenging promises of digital health and negotiating health expertise. In the following sections, we will present the findings of this study. The quotes shown in the following sections have been pseudonymised to ensure the anonymity of the interviewees.

The Finnish study was conducted according to the guidelines of the Finnish National Board on Research Integrity TENK (2019), the Swedish study with approval from the Swedish Ethical Review Authority (Approval No. 2022‐05780‐01) and the Danish study was approved by a Research and Innovation Organisation (RIO; Journal No. 11638).

## 5 Aiming for Self‐Responsibilisation

The prevailing expectation that citizens must be proactive and self‐responsible in maintaining their health and preventing health problems was present in many interviews. For some, it meant following health providers' instructions conscientiously, as was the case with this interviewee:

If I go to the doctor and he tells me that I need [to do] this and that, I always try to do it when I'm told, and this way, if it would help me, because no one else will help me but myself.

(INT_51, a 79‐year‐old man)

There were also instances when the interviewees were compelled to be self‐directed and active because they felt that healthcare had somehow failed them. One male interviewee (INT_53), for instance, went to see a doctor due to neuralgia in his leg. He, however, felt that the doctor did not give him a referral to surgery due to his high age (88 years). Consequently, the interviewee had no other option but to figure out himself how to ease the pain. According to him, he started searching for information on neuralgia treatments online and ended up finding stretching exercise instructions that helped him significantly with his ailment. In a similar vein, another interviewee told us that he did not trust the health clinic to take his blood pressure and mentioned self‐tracking and being active in one's health management as a way to guard himself against the incompetence of the healthcare system:

I check everything. If they [healthcare staff] say something, or think something's missing, I'll add it. But you have to constantly be pushing and advocating, and speak up yourself, otherwise they can say ‘it was like this and that, the tests said this, goodbye’.

(INT_11, a 77‐year‐old man)

The extract above conveys that to receive needed treatment or care from healthcare providers, one must be able to express and exercise agency in a way not all citizens are capable of. What is required is not only a basic level of health literacy and communication skills but also the confidence to advocate for oneself within a complex healthcare system. If one lacks the necessary skills, knowledge or confidence to navigate these challenges effectively, as many of our interviewees did, individuals are left at a disadvantage when it comes to accessing appropriate care, inevitably endangering their right to equal health.

As healthcare systems are facing challenges in providing adequate services for their citizens, individuals are not only expected to be active and responsible for their own health but for the health of others too (see Petrakaki et al. 2021, 2). Exemplifying a ‘good’ citizen in this regard was one male interviewee, aged 80, who was recovering from a heart bypass surgery operation. He told us that:

When I was recovering from bypass surgery, and I didn't really get any kind of clues [of what my heart health status was]. I had to figure out myself how to go on with my life. Then I thought that I have to pull myself together and try to help others who are in a similar situation, no matter what kind of medical operation it is.

(INT_48)

The interviewee then started organising group exercise classes for the members of a local heart association, where each participant's heart rate was measured. After the class, he would either email or give a printed version of the heart rate graph to each participant and explain to them how they could utilise the data to advance their own heart health. The same interviewee also had a central role when the association organised an informational webinar on coronary artery disease; according to him, he took care of the live streaming of the event so that people who were not able to come on site could still participate and learn more about the disease and related self‐care.

## 6 Utilising Digital Health Technology for Health/Risk‐Management

Many of our interviewees used some digital technology for health purposes, primarily step counters on smartphones or smartwatches. Some also used blood pressure and heart rate monitors at home. Although step counters allowed our interviewees to track their movement and exercise, devices such as blood pressure monitors allowed them to tangibly monitor and manage their health by taking charge of the measuring that usually happens in the doctor's office. This kind of measurement can be seen as a direct implementation of the self‐care and self‐responsibilisation urged within digital health citizenship (Petrakaki et al. 2021).

For some individuals, a heart rate and blood pressure monitor allowed them to manage life‐threatening risks after intense health events. As the 80‐year‐old man, who after a heart bypass operation started carefully measuring and analysing his heart rate graphs, described:

That was one of the ways I got through that recovery after the bypass surgery on my own, where I was always trying to get these measurements as early as possible, to get these measurements on, so that I could first of all maintain a heart rate level that didn't interfere with the healing that was needed for recovery. Because if I had strained myself too much right away, there might have been some problems during the healing phase.

(INT_48)

Here, digital health technology awarded him some freedom in managing his condition by ensuring that he did not overexert himself and jeopardise his recovery. It is also an example of the digital health citizenship imperative for individuals to generate health data and become experts in their medical conditions, consequently turning citizens into ‘diagnostic agents’ who are expected to do the work traditionally performed by healthcare professionals (see Oudshoorn 2008).

During the height of the COVID‐19 pandemic, there was significant discussion in the media about how one, without knowing, could have low oxygen levels, which could be a sign of severe COVID‐19. One interviewee (INT_11, a 77‐year‐old man) told us that he had purchased a pulse oximeter to make sure he would not unknowingly have ‘unhealthy’ rates of oxygen in his blood and a possible COVID‐19 infection. Here, the health technology allowed the interviewee to generate health data that he could not ‘feel’ on his own, and once generated, could impact how he acted—that is, avoiding certain activity if it resulted in lower oxygen levels. Another interviewee, a 75‐year‐old man (INT_34) with a chronic pulmonary disease, also monitored his oxygen level through a pulse oximeter. He would measure his levels, especially during physical training, and then use the reading to explain how he was feeling:

I have one of the exercises where I stand up here and then lie down again. And when I've done that five times, I'm done. And then the air, completely down to 80. And then I can just sit still for 5 minutes, and it's up to 92. Maybe 93. […] Well, it's just very reassuring to know. Sometimes when I feel really bad, I think, ‘Who knows?’ and then I put it on, and it's damn well below 80. Then it's right down at the critical level.

(INT_34)

The oxygen monitor here became a reassuring tool that confirmed the interviewee's felt sense of being ‘out of air’. He could then rest and see how the levels were slowly elevated up to a healthy level again, without having to take more drastic measures such as going to the emergency room. This is an example of trusting technology more than one's own body and relying on it to interpret physical experiences. In a way, the interviewee handed himself over to the technology instead of relying on his own feelings and experiences (see also Bavngaard 2023).

Using digital health technologies to monitor chronic illnesses was something several interviewees talked about. This was expected as monitoring and measuring blood pressure and cholesterol levels have long been part of older adults' health‐related risk management, due to chronic cardiac conditions often becoming more common in older age. One 77‐year‐old male interviewee (INT_11) was particularly self‐managerial with his blood pressure measuring. He explained that taking the blood pressure at the health clinic took too long because *you'll only be ignored, and you'll have to wait for them to call you back*. Therefore, instead of trusting the health clinic, he chose to take his own blood pressure and mark the measurements in his Google calendar, which he would then show to the doctor. The interviewee also argued for the advantage of home measuring by explaining that he would often come to the doctor in a stressed‐out state which, according to him, led to an unnaturally high blood pressure that was not accurate.

Similarly, a 79‐year‐old male interviewee (INT_14) took his blood pressure at home monthly and recorded it in a document together with notes from his doctor's appointments. He described one specific instance of having been prescribed blood pressure medication because a measurement at the doctor's office had been abnormally high. Because he was not feeling well on the medication, he started recording his blood pressure at home, which eventually enabled him to stop taking the medication after telling his doctor about the lower measures he had recorded. Both cases (INT_11 and INT_14) are examples of successful health self‐management where the individuals' resourcefulness at home allowed them to navigate a strained healthcare system. When the healthcare provider failed to adequately measure and monitor their blood pressure, these individuals stepped in and took responsibility for their own health conditions. This works well for the resourceful older adults mentioned here but not for those who lack the skills, knowledge or confidence to navigate digital health technologies and the general self‐management of health.

Among some of the savvier digital health technology users who actively engaged with different health tools, some did so with the option of sending measurements directly to their doctors. One of them, a 75‐year‐old woman (INT_65), had a blood sugar tracker and blood pressure meter that were connected to her smartphone, from which the measurements could be sent to a doctor. Despite this function, the interviewee herself kept track of the various measurements and altered her behaviour if needed. Additionally, she used a pill reminder application on her smartphone to manage multiple medications and an application to monitor heart rhythm and atrial fibrillation, exemplifying great self‐managerial skills and a successful enactment of digital health citizenship (Petrakaki et al. 2021) in this regard.

The same interviewee (INT_65) also told us that she used a fitness watch to help her optimise the efficiency of her daily water exercise and played Tetris on her smartphone *to keep dementia at bay*. If cognitive training, such as, playing Tetris or Sudoku on digital devices, was perceived as helping in controlling dementia risk, some interviewees were also clearly aware of alleged health risks related to physical inactivity in later life (see, e.g., Tulle 2015). This had motivated some individuals to use a wearable tracker or smartwatch to help them maintain a certain activity level:

You bet I care [about step counting]. Before the surgery I used to walk 10.000 steps each day. Now it is about half […] I keep track. […] If I haven't reached 5000, then I have to walk a little longer.

(INT_44, an 80‐year‐old man)

If I don't get many steps, the next day I must go for a walk. That's how it goes for me.

(INT_59, a 78‐year‐old woman)

These ‘have‐to's’ and ‘must‐go's’ in relation to the step counter numbers echo the normative expectations embedded in health citizenship that exhort individuals to be proactive and engaged with one's health maintenance (see also Spoel et al. 2014). These individuals had internalised the imperatives of digital health citizenship, which directly influenced their behaviours. However, this ‘neo‐liberal imperative to avoid inactivity’ (Katz and Marshall 2018, 67) also aroused resistance among some older citizens as they wanted to cherish a less active lifestyle after retirement. After having to go to work for most of their lives, some wanted to hold on to their autonomy and *not* do things that society expects them to do—despite it being *bad for yourself when you don't* *do it*, as one 75‐year‐old woman (INT_49) explained.

## 7 Challenging Promises of Digital Health

The use of blood pressure monitors, pulse oximeters and step counters described above are all examples of normative enactments of digital health citizenship. These interviewees found digital health technologies useful in managing their health and potential risks and were able to navigate shortcomings of healthcare systems by undertaking measures that previously were only available in doctors' offices. However, there were also individuals who had a more ambivalent or even resistant stance on, for instance, tracking being helpful in achieving better health. It was acknowledged that in order to truly benefit from personal health technologies, one needs to be engaged with their use and actively examine the data. As one interviewee with a smart ring tracking physical activity, heart rate and sleep explained:

This only just registers what happens in the body. You have to take the measures yourself. (…) And you can print out the reports here. Then you should analyse them and think about them.

(INT_54, a 75‐year‐old man)

The interviewee told us that he checked the smart ring data every morning but admitted having difficulties comprehending the readings and making use of them—a tendency that many of our interviewees seemed to share. There were a few exceptions to this, like the interviewees with a professional medical background, who were confident in their ability to interpret the available health data for them. Apart from these older citizens, tracker readings along with online health records were often checked merely out of interest rather than with the intention of using them for personal health management purposes, as presumed in the ideal formulations of digital health citizenship (Petrakaki et al. 2021).

The current policy expectation that individuals should use different digital platforms for appointment booking, symptom assessment and communication with healthcare was something many of our interviewees struggled with. Some did not even have the required devices or skills to use digital services. However, most interviewees were aware of the key online health portal in each country, and many had also used it at least once, for instance, to check laboratory test results or prescriptions. Our interviewees rarely renewed their prescriptions digitally, though, but tended to ask a pharmacist or a health provider to do it. In addition, several interviewees expressed relatively low levels of enthusiasm about using online health portals, as exemplified by the following excerpts:

I've been there [MyKanta.fi] a few times, but I don't really understand it. I don't know how to go there, and I don't know what the doctors write down. I'm not interested at all what they write there.

(INT_68, a 75‐year‐old woman)

No, I haven't really [read my own file online]. I assume that the conversation I have with the doctor before I finish the consultation covers it. So there's no reason to sit and flip through it. And half of it is in Latin, which I don't understand a thing of.

(INT_42, an 84‐year‐old man)

For these individuals, the information available online was a source of confusion, noninterest or irritation. As the second quote above reveals, the direct meeting with the doctor and the information conveyed there weighed much heavier than (presumed) hard‐to‐parse medical jargon. In addition, the prevailing expectation that one should use digital platforms and available data in one's health management was challenged—instead, some interviewees chose to opt out of using digital provision:

If I need a doctor, (…) I go through the city centre, and I can just easily book an appointment [at the health center] or ask when I can get it, or I can call by phone, either one. Those are my channels.

(INT_62, an 80‐year‐old woman)

Some of our interviewees also implied that personal digital technologies were not very useful in enhancing self‐knowledge or health improvement initiatives: *the steps don't tell me anything*, as one interviewee argued. Two other individuals, respectively, described counting steps as *superfluous information* and even *enslaving* because it made people constantly check their trackers. Instead, trusting one's self‐assessment and listening to one's own bodily sensations was considered as being enough (cf. Bavngaard 2023):

I guess you know roughly how much you move. And then sleep, you don't need a smart device for that, do you. I don't care much about them. But even smart people have them [laughs]. Yeah, it never occurred to me that I'd get one.

(INT_67, a 75‐year‐old woman)

I've noticed in the gym, they have a thing that they press the button to see how many calories have now gone and how the heart has now beaten and so on […] but I'm so old that I no longer indulge in such things, I know when I start to sweat and when I feel good and bad.

(INT_52, a 79‐year‐old man)

Here, data generated by digital technologies were not seen as providing a way of (better) knowing and understanding one's body, which runs counter to the premise of data‐driven knowledge providing enhanced possibilities to manage personal health embedded in the notion of digital health citizenship. Some interviewees were also sceptical about whether the used technology could be trusted, as the following excerpts indicate:

[The step counter] is not really reliable. One time I had gone up and down those stairs three times and it only showed once.

(INT_7, an 88‐year‐old woman)

I think it's lying [the smart watch]. Even now, it reads 86. And it's not that much. If I take the blood pressure gauge and measure it, it's probably not 86. I don't really know which device to believe.

(INT_49, a 75‐year‐old woman)

Additionally, many of our interviewees expressed a lack of interest in using and purchasing digital health tools. Several of the interviewees with smartphones mentioned that they knew their phones came with a ready‐installed health application, but they were not interested in using it. This lack of interest often did not have any clear motivation; it merely was not relevant to their lives, and they, therefore, chose not to use these health applications (see also Bagger et al. 2023; Helsper and Reisdorf 2013).

## 8 Negotiating Health Expertise

Developing expertise in one's own health/illness condition is positioned as a key aspect of health citizenship and digital health citizenship (Jauho and Helén 2023; Petrakaki et al. 2021). The individual is urged to obtain sophisticated knowledge about her/his own condition so that they can act responsibly to maintain good health. Some of our interviewees aimed for expertise in their health condition by trying to take in knowledge provided by health providers and wanting to read *everything what they've [doctors] written about me* (INT_63, a 75‐year‐old woman). However, this kind of dedication was not as common as more occasional online information searches on topical illnesses and ailments one had then. Sometimes also a desire to know more about a prescribed medicine or an upcoming medical procedure motivated our interviewees to seek knowledge online. As one digitally savvy interviewee said, *and* *then you Google quite a lot about any illness you have* (INT_15, a 77‐year‐old woman).

Several of our interviewees, however, expressed reluctance towards searching for information about their health issues on platforms such as Google. There seemed to be a fairly widespread idea about googling leading to worry and even wrongful self‐diagnoses:

I Google. From there, I've read about so many diseases from the beginning to the end (…) But then again, with my heart, I'm getting more and more scared. Of course, knowledge is knowledge and it's good to know, but then I have this, and I have that, and (…) I feel like death is near [laughs].

(INT_49, a 75‐year‐old woman)

There were also a few individuals who knew where to find reliable and science‐based information online. One interviewee told us that he would always go first to the national medical society's website if he had a health‐ or illness‐related question in mind:

From there I'll see if there's any illness, and I'll look into what it could be and what I should do, and so forth.

(INT_58, a 77‐year‐old man)

The ones who were not so familiar with the internet and digital technology tended to rely on TV programmes, newspapers, magazines and senior organisations for health information. Tips for self‐care and healthy diets, and experiential knowledge on illnesses and medication were also shared within one's social circles, whereas participation in health‐related online support groups and the like was very rare. In fact, most of our interviewees found the whole idea of online peer‐to‐peer interaction and personal health experience‐sharing rather alien or even amusing. Consequently, the value or benefit of peer support and community building via digital media (cf. Petrakaki et al. 2021, 5) was perceived as minimal:

People are differently ill and experience their illness differently than I do. So I have no need to go online, and I don't read anything there what is said about osteoarthritis, what the different forums say about it. No.

(INT_66, a 78‐year‐old woman)

Although becoming an ‘informed citizen’ is positioned as central in formulations of health citizenship, having a good knowledge of health and illness does not automatically mean that one will live and eat healthily: *it all depends on yourself*, as one retired medical doctor (INT_67, a 75‐year‐old woman) vigorously noted. The preceding quote also implies that healthy living is merely a matter of individual choice and willpower, which ignores the complexity of people's lives, health situations and socioeconomic circumstances.

When the interviewees were asked how they took care of their health, some individuals stated that they were not particularly proactive with it, as the next quote indicates:

In theory I know everything I should do. I've had one gym session. It's one hour a week, but no. I try to eat sensibly, meaning, I try to remember to eat.

(INT_56, a 77‐year‐old man)

The perception of what a healthy lifestyle consists of and how to pursue it naturally varied from one individual to another. One 85‐year‐old woman (INT_1) said that she tries to live healthily but also *wants to live well* and treat herself to wine, for instance, when dining. For several other interviewees too, maintaining good health was not just about engaging in a healthy and physically active lifestyle. As one male interviewee (INT_58, aged 77) explained, a good marriage accompanied by a relatively worry‐free and privileged life had helped him in ageing healthily. Interviewees with full social calendars noted that maintaining an active social network was very important to their overall health and well‐being. These examples, in particular, highlight the important role that social relations and socioeconomic circumstances play in maintaining health and well‐being in later life, aspects that are not included in the normative formulations of digital health citizenship.

## 9 Concluding Discussion

To the best of our knowledge, the concept of digital health citizenship has not been explored in the context of aged individuals before our study. This study represents an effort to understand how these individuals engage with digital health technologies and what this means for their expected role as digital citizens within the healthcare system. We explored the complexities and nuances that arise from normative expectations of digital health citizenship (Petrakaki et al. 2021) in late life under the following four themes that emerged from the data: *aiming for self‐responsibilisation*, *utilising digital health technologies for health/risk management*, *challenging promises of digital health* and *negotiating health expertise*.

The expectation of self‐responsibilisation in digital health citizenship surfaced in examples where the individuals tracked their daily steps, used a pulse oximeter or measured their own blood pressure for health‐related risk management. For these interviewees, self‐tracking at home served them well, in that it allowed them to have more say in their own treatment and catch things early. As such, it provided transparency into their own health and enabled personal risk management, for example, more steps than calling a doctor (Ruckenstein and Pantzar 2017).

Previous studies have shown that individuals use technologies differently and in ways that fit their own routines and goals (see, e.g., Pols and Willems 2011). In our study, this became apparent, for instance, when an interviewee preferred at‐home blood pressure measuring to avoid ‘unnaturally’ high readings at the health clinic and marked the measurements in his Google calendar instead of using an online platform provided by the public healthcare. Or, when an interviewee played Tetris on her smartphone because she saw this practice as supporting her brain health—further illustrating that uses and interpretations of digital technologies are also contingent, as are the claims about their effects and capacities (Marent and Henwood 2023, 41).

Broadly speaking, digital media, such as smartphone applications and wearable technologies, have enabled new means of self‐tracking and health management and made digital health systems available for more people (Rooksby et al. 2014, 1163). In our sample, both men and women who were tech‐savvier had used computers and the internet in work life and were often better educated, which resonates with existing studies (see, e.g., Hunsaker and Hargittai 2018). Male interviewees belonging to this tech‐savvier group also talked at greater length about their use of digital health technologies and related applications, often expressing a long‐term interest in technology, which previous studies have found to predict more likely use of health‐related applications and devices (see Seifert and Vendelanotte 2021).

Some interviewees made quite an effort to keep personal health records and seek expertise in their own health. This approach served as a form of reassurance against the shortcomings of the healthcare system or as a response to a lack of trust in that system. By maintaining their own records, individuals assert a level of control over their health information, ensuring that critical data are accurate, complete and readily accessible (Lupton 2016). This practice can be particularly important in situations where patients feel that the healthcare system may be prone to errors, omissions or inefficiencies (see Nissenbaum 2009). Moreover, keeping personal records can be seen as reflecting a proactive approach to health management, embodying the responsibilities of digital health citizenship (Petrakaki et al. 2021). It allows individuals to cross‐check medical advice, track their health over time and make informed decisions, thereby mitigating the risks associated with system failures.

However, not all citizens are able or willing to be so self‐responsible and proactive to actively use digital health. Even though some individuals in our study used self‐tracking services and benefited from them, many expressed resistance towards accessing health digitally and using self‐tracking technology. Several of the oldest interviewees also lacked the skills, confidence, resources and/or interest to use digital health. Our data also showed that it is often difficult for individuals to interpret the health information generated by digital health technologies and that there can be distrust in the monitor metrics and applications. Such challenges can lower one's interest in engaging with digital health tools and reduce opportunities to enact digital health citizenship, the ways expected in contemporary health policies. At the same time, refusal to use health technologies and be self‐responsible is one way of exercising agency and of resisting the stereotype of being at risk due to higher age (see Aceros et al. 2015).

Studies have shown that many older adults often face various challenges when using digital health (Varjakoski and Tiilikainen 2025), which directly hinder their digital engagement with healthcare. This includes, for instance, difficulties in using digital health services due to poor interface usability and accessibility, along with age‐related physical and cognitive changes that can prevent engagement with digital health technologies (see also Han et al. 2015). For instance, a few of our interviewees had very bad eyesight, almost at the level of blindness. Even if they had the skills, confidence and interest to use digital health technology, they would not be physically able to do it by themselves. For these individuals, digital health citizenship would only be achievable through other people, as was the case with some of our interviewees too. Help was needed to use technologies, understand complex health systems and gain confidence in one's ability to navigate them (see also Hänninen et al. 2021; Søgaard Nielsen and Grønning 2025). However, the need for help from others may lead individuals to reluctantly share personal information with family members or even avoid certain health services altogether (Søgaard Nielsen 2025). This is an apt reminder that technology use and the ability to manage one's health are often deeply ‘entangled with the relations, affectivity and complexities’ of people's lives, as Rich and Lupton (2022, 6) argue.

A key component of digital health citizenship, as defined by Petrakaki et al. (2021), is the requirement to share self‐collected health data with healthcare providers, highlighting how such contributions can, for instance, advance disease research and help identify gaps in clinical practice. Another aspect of this is sharing health data in peer‐to‐peer online networks for support and knowledge exchange. However, the older adults in our study did not really engage in this kind of digital production of knowledge. This disengagement can limit the ability to influence healthcare practices and research and result in the experiences and insights of older people not being represented in broader digital health discussions and development. Additionally, individuals who do not use digital devices and the internet often have very limited opportunities to provide feedback to healthcare providers, as most client surveys and feedback forms are now available only online. This further excludes certain individuals from their right as citizens to have their voices heard in health policymaking and in the organisation of health services.

It can be argued that digital health citizenship was achievable for those interviewees who were digitally savvy and had incorporated digital health measurements into their everyday lives. However, this was not the case for a large group of our interviewees who, for distinct reasons, did not (or could not) use digital health, relied on their own body knowledge instead of digital data, obtained health information from nondigital sources and/or adhered to traditional provisions of healthcare, as these worked better for them. Concurrently, this often also meant that their right as citizens to accessible and equitable healthcare, for instance, was potentially compromised by the vast digitalisation of health. Overall, in our study, the accomplishment of digital health citizenship in late life appeared selective and fluctuating, depending on the person's health status, among others. For some interviewees, the accomplishment was often also relational, which challenges the existing individualist theorisations of digital health citizenship.

Our study managed to reach individuals and experiences that digital health research often fails to capture, that is, individuals who are representatives of the oldest demographic, have lower digital skills and poorer physical functioning (Poli et al. 2020). Overall, we were able to gain a deep and contextualised understanding of digital health practices and engagements of older individuals in this study. This study has implications for policy and practice within and beyond the Nordic countries. As developed countries increasingly digitalise healthcare and urge individuals to take responsibility for their own health, the findings highlight the importance of recognising the diverse capacities and resources of people to access, use and benefit from digital health. As implications for practice, the findings indicate the need to pay more attention to relationality in digital health practices and to secure nondigital healthcare provision for individuals at risk of digital exclusion, such as people with severe physical disabilities, cognitive decline or multimorbidity.

## Author Contributions

**Hanna Varjakoski:** conceptualization (equal), formal analysis (equal), investigation (equal), methodology (equal), writing – original draft (lead), writing – review and editing (lead). **Fredrika Thelandersson:** conceptualization (equal), formal analysis (equal), investigation (equal), methodology (equal), writing – original draft (equal), writing – review and editing (supporting). **Amalie Søgaard Nielsen:** conceptualization (equal), formal analysis (equal), investigation (equal), methodology (equal), writing – original draft (equal), writing – review and editing (supporting).

## Funding

This work was supported by the Kamprad Family Foundation, via SLS in Finland and the Future Challenges in the Nordics research programme (Grant No. 20212004).

## Ethics Statement

The research has been ethically approved in each country.

## Data Availability

Access to the data is maintained by the HAIDI research team. Requests to access these datasets should be directed to Helena Sandberg (helena.sandberg@iko.lu.se).
